# Effects of liraglutide or lifestyle interventions combined with other antidiabetic drugs on abdominal fat distribution in people with obesity and type 2 diabetes mellitus evaluated by the energy spectrum ct: A prospective randomized controlled study

**DOI:** 10.3389/fendo.2022.951570

**Published:** 2022-08-26

**Authors:** Dongni Yu, Mingzhu Zou, Qi Pan, Yan Song, Miao Li, Xianbo Zhang, Yan Zhou, Xiaoxia Wang, Lixin Guo

**Affiliations:** ^1^ Department of Endocrinology, Beijing Hospital, National Center of Gerontology, Institute of Geriatric Medicine, Chinese Academy of Medical Sciences, Beijing, China; ^2^ Department of Radiology, Beijing Hospital, National Center of Gerontology, Institute of Geriatric Medicine, Chinese Academy of Medical Sciences, Beijing, China

**Keywords:** liraglutide, obesity type 2 diabetes mellitus, abdominal fat distribution, type 2 diabetes, diabetes

## Abstract

**Objective:**

To study the effects of liraglutide or lifestyle interventions combined with other antidiabetic drugs on glucose metabolism and abdominal fat distribution in patients with obesity and type 2 diabetes mellitus (T2DM).

**Methods:**

From April 30, 2020, to April 30, 2022, a prospective randomized controlled study was carried out at the Endocrinology Department of Beijing Hospital, the National Center of Gerontology. According to the in- and exclusion criteria and by the random table method, revisited T2DM patients were selected as the research subjects and were allocated into a Study group (taking liraglutide) and a Control group (underwent lifestyle interventions). All patients received continuous 12-weeks interventions to the endpoint, and the changes of value [Δ=(endpoint)-(baseline)] of physical measurements, blood tests, the energy spectrum CT examination results, and body composition analysis results were analyzed and compared.

**Results:**

A total of 85 people completed this study, and among them, 47 were in the Study group and 38 were in the Control group. Compared with the Control group, the changes of hemoglobin A1c (HbA1c) level (-0.78 ± 1.03% vs. -1.57 ± 2.00%, *P*=0.025), visceral fat area (0.91 ± 16.59 cm^2^ vs. -7.1 ± 10.17 cm^2^, *P*=0.011), and subcutaneous fat area of abdomen [0 (-18.75, 15.5) cm^2^ vs. -16.5 (-41.75, -2.25) cm^2^, *P*=0.014] were all greater in the Study group. The adverse events caused by liraglutide were mainly concentrated in the gastrointestinal system and all of them were minor adverse events.

**Conclusion:**

Liraglutide can be the drug of choice for weight management and reduction of abdominal fat distribution in patients with obesity and T2DM.

## Introduction

With the development of the economy and changes in lifestyles, the prevalence of type 2 diabetes mellitus (T2DM) has risen rapidly; the latest data from the International Diabetes Federation (IDF) shows that the global prevalence of T2DM in adults will be as high as 10.5% in 2021, and it is predicted that it would be risen to 12.2% in 2045 (1). The proportion of overweight and patients with obesity and diabetes is high, and obesity is closely related to diabetes (2). According to the results of the World Health Organization (WHO) survey in 2013, obesity accounts for 45.8% of patients with T2DM (3). Ectopic fat deposition can lead to pathological changes such as insulin resistance and chronic inflammation, and studies have confirmed that excessive deposition of abdominal fat, especially intra-abdominal fat, is closely related to cardiovascular disease and diabetic nephropathy in diabetic patients (4). The energy spectrum CT technology that appeared in recent years has the characteristics of rapidity, high accuracy, and good repeatability in the quantitative examination of abdominal fat, and the results are in good agreement with the methods of Magnetic Resonance Imaging (MRI) and Dual Energy X-ray Absorptiometry (DEXA), which can make it more convenient to evaluate the changes in abdominal fat and provide more useful clinical information for physicians and patients to control the disease process of the T2DM (5).

Glucagon-like peptide-1 (GLP-1) receptor agonist liraglutide is a new type of hypoglycemic drug, which can control blood sugar, help lose weight and reduce the risk of cardiovascular disease; it has become the first injection choice for type 2 diabetes for antidiabetic drugs according to guidelines at home and abroad (6). However, there are inconsistent results on the effects of GLP-1 receptor agonists on the changes in body composition in obesity and diabetes (7), and there is a lack of clinical studies to observe effects of GLP-1 receptor agonists on abdominal fat distribution.

This study aims to study the effects of liraglutide or lifestyle interventions combined with other antidiabetic drugs on metabolism and abdominal fat distribution in patients with obesity and T2DM.

## Methods and material

### Study design

**Figure 1:**
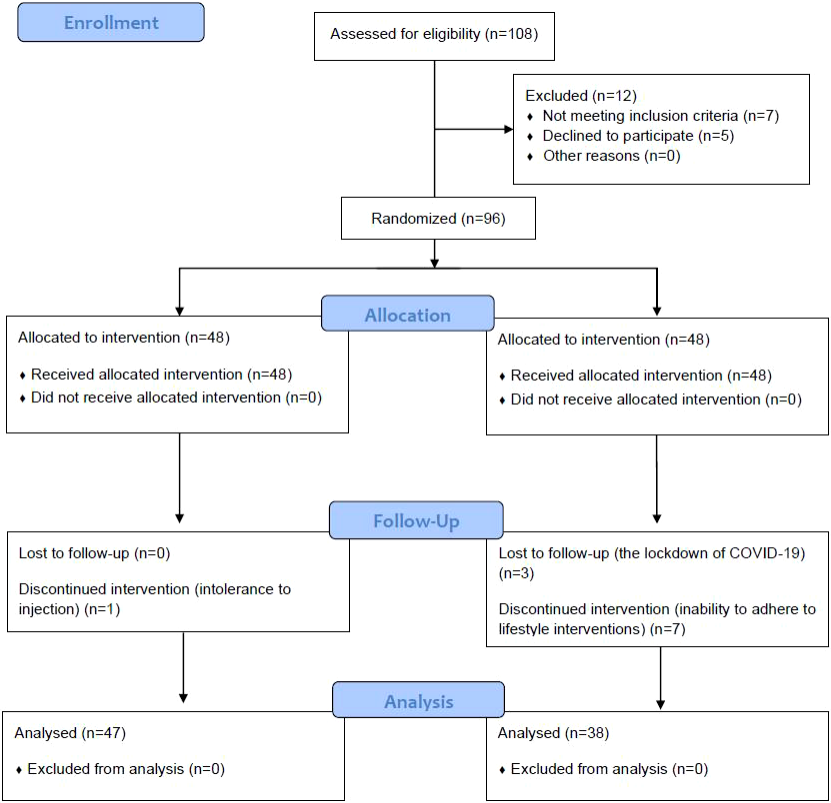
CONSORT Flow Diagram.

From April 30, 2020, to April 30, 2022, T2DM patients in an outpatient clinic at the Endocrinology Department of Beijing Hospital, the National Center of Gerontology, were selected as the research subjects. According to the in- and exclusion criteria and by the random table method, patients who voluntarily participated in this study and signed informed consent had been allocated into two groups, a Study group and a Control group, and a prospective randomized controlled study was carried out. The enrollment process was shown in (**Figure 1**).

This study was approved by the Ethics Committee of the Beijing Hospital, the National Center of Gerontology, with the ethics approval number 2019BJYYEC-254-02. This study had been registered in the Chinese Clinical Trial Registration Center with the registration number ChiCTR2000031943.

### In-/exclusion criteria

Inclusion criteria were as follows: 1) Patients Aged between 18 and 65; 2) Patients diagnosed with type 2 diabetes in the past (8); 3) Body Mass Index (BMI)≥ 28kg/m^2^; 4) hemoglobin A1c (HbA1c) ≥ 6.5% at the time of follow-up; 5) Those who had been treated with hypoglycemic drugs and had not undergone lifestyle interventions; 6) Accepted any one of the two intervention methods and signed the informed consent.

Exclusion criteria were as follows: 1) Patients were diagnosed with other types of diabetes, such as type 1 diabetes, special type of diabetes, etc.; 2) Patients with severe acute and chronic complications of diabetes, such as hyperosmolar coma, diabetic ketoacidosis, diabetic eye disease, diabetic nephropathy, and diabetic peripheral neuropathy; 3) Dipeptidyl peptidase 4 (DPP-4) inhibitor had been used in the past 3 months; 4) Combined with severe liver function damage (ALT≥120U/L); 5) Combined with severe renal insufficiency; 6) Combined with severe heart failure [New York Heart Association (NYHA) Classification: III-IV]; 7) Combined with severe hypertriglyceridemia [Triglyceride (TG) ≥5mmol/L]; 8) Those with mental disorders who cannot cooperate with the experiment; 9) Pregnant and lactating women; 10) Patients with definite diagnosis of secondary obesity, such as hypothyroidism, Cushing’s syndrome, etc.; 11) Long-term use of drugs that can affect the weight (such as orlistat, glucocorticoids, etc.); 12) Alcoholism, the ethanol intake is more than 140 g/week for men and more than 70 g/week for women; 13) Patients with high risk factors of medullary thyroid cancer and abnormal thyroid function; 14) History of tumor within 5 years; 15) Patients who cannot strictly comply with the intervention program; 16) Those who are allergic to liraglutide and its excipients.

### Study method

The patients who agreed to participate in this study signed the informed consent form, and then, questionnaires, physical measurements, blood tests, the energy spectrum CT examination, and body composition analysis were completed to obtain the baseline data.

Based on the patients’ original hypoglycemic treatment plan, the patients in the Study group were given liraglutide treatment; patients in the Control group were given lifestyle interventions accordingly. In the Control group, a diet plan was formulated by a nutritionist. Based on each person’s target energy intake, the daily energy intake was reduced by 500-1000 kcal, and finally, the daily energy intake of male patients with obesity was 1200-4000 kcal, and the daily energy intake of female patients with obesity was 1000-1200 kcal, of which carbohydrate accounts for 55-60% of total daily energy, and fat accounts for 25-30% of total daily energy. All patients in the control group were required to perform at least 30 minutes of moderate-intensity aerobic exercise five days a week. In the Study group, patients received once-daily weight-based injections of liraglutide. The two groups of patients received continuous intervention for 12 weeks. After 12 weeks, the patients were at the endpoint and were given the questionnaire, physical measurement, blood tests, the energy spectrum CT examination, and body composition analysis again. The change value of related indicators before and after the intervention, [Δ=(endpoint)-(baseline)], was recorded and analyzed.

### Observation indicators and adverse events

#### 1) Basic information collection

The basic information of the patients was collected through questionnaires as follows: duration of T2DM, the combination of other diseases, current T2DM treatment protocol (types and doses of hypoglycemic drugs), disease history, personal history, and female menstrual history.

#### 2) Physical measurements

After fasting for one night and the bladder was emptied, patients were under the physical measurements of height, weight, waist circumference, and hip circumference, and then, the BMI (kg/m^2^) and waist-to-hip ratio were calculated. All the physical measurements were carried out by the same trained investigator,

#### 3) Blood test

The patients have fasted for more than 8 hours, and venous blood was drawn for the test of fasting blood glucose, and biochemical indicators [urinary albumin excretion rates (UAER), creatinine (CRE), Blood urea nitrogen (BUN), Aspartate aminotransferase (AST), Alanine transaminase (ALT), and HbA1c].

#### 4) Energy spectrum CT examination

Firstly, patients were in the supine position and underwent the energy spectrum CT examination, the energy spectrum CT (Revolution 256, GE, United States) was used, the energy spectrum mode was set for scanning, and the scanning range was from the top of the diaphragm to the level of the pubic symphysis.

**Figure 2:**
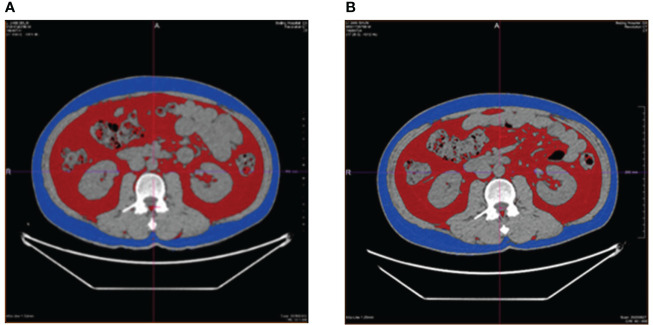
Before-and-after comparison of visceral fat area, the red area, and the subcutaneous fat area of abdomen, the blue area, in a patient receiving liraglutide intervention. **(A)**, before the intervention of liraglutide; **(B)**, after the intervention of liraglutide.

Subsequently, fat (relative to muscle) density images and muscle (relative to fat) density images were obtained using GSI software and under the condition of 70keV single energy. The L3 vertebral transverse process level was selected for measurements of the energy spectrum CT examination, and the measured data were as follows: abdominal cross-sectional area and subcutaneous fat area of abdomen; liver fat content; abdominal subcutaneous fat thickness; back subcutaneous fat thickness; subcutaneous fat thickness outside erector spine; Erector spinae intramuscular fat content. (**Figure 2**.)

The energy spectrum CT results were analyzed and reported by two independent radiologists, who did not know the grouping of the patients. If the interpretation results were inconsistent, the dean of the radiology department would make the final data analysis.

#### 5) Body composition analysis

Patients were dressed in light and thin clothes, without carrying metal objects, stood barefoot on the body composition meter (Lookinbody720), and the electrical impedance method was used to conduct body composition examination to obtain data such as body mass, skeletal muscle, body fat, and visceral fat area. Body fat percentage (%) was calculated as follows: Body fat percentage (%) = body fat (kg)/body weight (kg) * 100.

#### 6) Adverse events

After 12 weeks of intervention, the adverse events were reported by patients in the Study group through questionnaires, such as nausea, constipation, and fatigue. The occurrence of hypoglycemia events was reported by patients in both groups.

Among all the observation indicators, the visceral fat area was taken as the primary outcome; the other indicators were regarded as secondary outcomes; and the adverse events were seen as other outcomes.

### Statistics

The primary outcome of this study was the visceral fat area. According to the similar study (9), the visceral fat area decreased to 49.6 cm^2^ in the Control group by lifestyle intervention and to 22.9 cm^2^ in the Study group after the continuous liraglutide injection, with a standard deviation of 40.0 in both groups. PASS 15.0 was taken to calculate the sample size, setting the parameters as α=0.05, β=0.2, and considering the dropout rate of 20%, a total of 94 subjects would be required for this study.

All the data collected in this study were analyzed using SPSS 22.0 software. Normally distributed measurement data were expressed as mean ± standard deviation (SD), while non-normally distributed measurement data were expressed as median (interquartile range), and the comparisons were examined by Student-t test and Mann-Whitney test (non-parametric distribution). The categorical data were expressed as n (%), and the differences between the two groups were examined by chi-square analysis or Fisher’s exact test. *P*<0.05 was considered statistically significant.

## Results

From April 30, 2020, to April 30, 2022, 108 patients were initially eligible and 12 patients were excluded (7 were not meeting the inclusion criteria and 5 declined to participate); finally, 96 patients who revisited the Endocrinology Department of Beijing Hospital, the National Center of Gerontology were selected as the research subjects according to the in- and exclusion criteria, and by the random table method, patients had been allocated into a Study group and a Control group, each group had 48 patients. In the Study group, 47 patients had completed this study and their data were analyzed, one patient cannot tolerant the injection and drop put halfway; and only 38 people in the Control group completed the study, with 3 patients dropping out halfway for the lockdown of their district and cannot complete their follow-up, with 7 patients cannot adhere to the lifestyle intervention (3 patients cannot keep working out and 4 patients were inability to control daily caloric intake as planned).

In the Study group, the proportion of female patients was 51.10%, the mean age was 51.53 ± 9.11 years, and the duration of diabetes was 2 (0, 6.25) years; correspondingly, in the Control group, the proportion of female patients was 65.80%, and the mean age was 49.97 ± 11.44 years, and the duration of diabetes was 3 (0, 8) years. There were no statistically significant differences between the above three data (*P*>0.05).

### Comparisons of hypoglycemic drugs used in two groups of patients

There were no significant differences between the two groups in the taking types of hypoglycemic drugs and the number of patients. (**Table 1**)

**Table 1:** Comparisons of hypoglycemic drugs used in two groups of patients.

Hypoglycemic drugs	Study group (n=47)	Control group (n=38)	*P* value
Metformin, n (%)	30 (63.80%)	21 (55.30%)	0.506
Acarbose, n (%)	16 (34.00%)	7 (18.40%)	0.142
Insulin secretagogues, n (%)	4 (8.50%)	2 (5.30%)	0.687
SGLT-2 inhibitors, n (%)	7 (14.90%)	10 (26.30%)	0.276
Insulin sensitizer, n (%)	2 (4.30%)	1 (2.60%)	>0.999
Insulin, n (%)	8 (17.00%)	4 (10.50%)	0.535
Liraglutide, n (%)			
0.6 mg	2 (4.30%)		
1.2 mg	16 (34.00%)		
1.8 mg	29 (61.70%)		

SGLT, Sodium-dependent glucose cotransporters.

### Comparisons and changes of blood sugar metabolism related indicators, physical measurements, and abdominal fat distribution

Comparing the endpoint levels and baseline levels of related indicators between the two groups of patients, it was found that the use of liraglutide reduced the fasting blood glucose level from 8.67 ± 2.98 mmol/L to 7.16 ± 1.53 mmol/L, *P*=0.004; the HbA1c level from 8.40 ± 1.83% to 6.83 ± 0.99%, *P*<0.001; the body weight from 87.27 ± 14.45 kg to 84.21 ± 13.75 kg, *P*<0.001; BMI from 31.12 ± 4.06 to 29.97 ± 3.60, *P*<0.001; waist circumference decreased from 105.98 ± 10.95 cm to 102.54 ± 9.13 cm, *P*=0.002; fat mass decreased from 32.52 ± 10.08 kg to 30.7 ± 9.00 kg, *P*<0.001; Abdominal cross-sectional area decreased from 368.16 ± 74.72 cm^2^ to 350.18 ± 82.03 cm^2^, *P*=0.016. Correspondingly, in the Control group, lifestyle interventions reduced fasting blood glucose levels from 8.39 ± 1.86 mmol/L to 7.31 ± 1.47 mmol/L, *P*=0.004; decreased the HbA1c level from 7.86 ± 0.83% to 7.08 ± 0.80%, *P*<0.001; body weight from 89.37 ± 14.71 kg to 87.54 ± 15.33 kg, *P*=0.006; decreased the BMI from 32.68 ± 3.80 to 32.02 ± 4.30, *P*=0.006; decreased waist circumference from 106.74 ± 9.72 cm to 104.12 ± 9.03cm, *P*=0.003; decreased fat mass from 35.82 ± 9.13 kg to 34.08 ± 9.61 kg, *P*=0.002; decreased Abdominal cross-sectional area from 374.72 ± 94.99 cm^2^ to 350.64 ± 97.85 cm^2^, *P*=0.007. Moreover, the liraglutide administration makes the body fat ratio decreased from 36.66 ± 6.96 to 35.42 ± 6.78, *P*<0.001; visceral fat area decreased from 131.50 ± 28.73 cm^2^ to 124.40 ± 28.68 cm^2^, *P*<0.001; liver fat content decreased from 7.05 (3.88,10.25) % to 4.18 (2.353,8.18) %, *P*=0.001. There were no statistically significant differences in other parameters between the two groups. (**Table 2**)

**Table 2:** Changes of blood sugar related indicators, physical measurements, and abdominal fat distribution.

Items	Study group (n=47)		*P* value	Control group (n=38)		*P* value
	Baseline	Endpoint		Baseline	Endpoint	
Fasting blood sugar (mmol/L)	8.67 ± 2.98	7.16 ± 1.53	0.004*	8.39 ± 1.86	7.31 ± 1.47	0.004*
HbA1c (%)	8.40 ± 1.83	6.83 ± 0.99	<0.001*	7.86 ± 0.83	7.08 ± 0.80	<0.001*
Weight (kg)	87.27 ± 14.45	84.21 ± 13.75	<0.001*	89.37 ± 14.71	87.54 ± 15.33	0.006*
BMI (kg/m^2^)	31.12 ± 4.06	29.97 ± 3.60	<0.001*	32.68 ± 3.80	32.02 ± 4.30	0.006*
Waist circumference (cm)	105.98 ± 10.95	102.54 ± 9.13	0.002*	106.74 ± 9.72	104.12 ± 9.03	0.003*
Hip circumference (cm)	110.99 ± 11.02	109.44 ± 8.32	0.141	111.53 ± 16.38	109.05 ± 18.87	0.518
Waist to Hip ratio	0.96 ± 0.06	0.94 ± 0.05	0.102	0.94 ± 0.07	0.93 ± 0.06	0.409
Fat mass (kg)	32.52 ± 10.08	30.7 ± 9.00	<0.001*	35.82 ± 9.13	34.08 ± 9.61	0.002*
Body fat ratio	36.66 ± 6.96	35.42 ± 6.78	<0.001*	39.95 ± 7.00	39.08 ± 7.09	0.107
Visceral fat area (cm^2^)	131.50 ± 28.73	124.40 ± 28.68	<0.001*	139.60 ± 26.67	140.51 ± 30.24	0.747
Abdominal cross-sectional area (cm^2^)	368.16 ± 74.72	350.18 ± 82.03	0.016*	374.72 ± 94.99	350.64 ± 97.85	0.007*
Subcutaneous fat area of abdomen (cm^2^)	221.50 (167.00, 310.00)	209 (161.75, 270.25)	<0.001*	230 (192.25, 319.75)	230.5 (196.00, 311.75)	0.689
Abdominal subcutaneous fat thickness (cm)	3.13 ± 1.18	3.03 ± 1.23	0.200	3.12 ± 1.02	3.16 ± 0.92	0.670
Back subcutaneous fat thickness (cm)	2.24 ± 0.93	2.20 ± 0.89	0.413	2.40 ± 0.75	2.41 ± 0.87	0.945
Subcutaneous fat thickness outside erector spinae (cm)	4.30 ± 1.35	4.15 ± 1.27	0.217	4.37 ± 1.06	4.36 ± 1.06	0.964
Erector spinae intramuscular fat content (mg/cm^3^)	61.50 (20.31, 124.05)	57.67 (1.05, 118.56)	0.843	52.14 (-25.80, 114.13)	43.86 (-9.44, 126.06)	0.405
Liver fat content (%)	7.05 (3.88, 10.25)	4.18 (2.35, 8.18)	0.001*	8.75 (3.89, 15.12)	6.37 (2.46, 14.32)	0.123

Data were expressed as mean± standard deviation and median (interquartile range).

HbA1c, hemoglobin A1c, BMI: Body Mass Index.

*Compared with the baseline level, P<0.05.

Further comparative analysis of the changes between the endpoint value and the baseline value between the two groups found that, compared with the Control group, the changes of HbA1c level (-0.78 ± 1.03% vs. -1.57 ± 2.00%, *P*=0.025), visceral fat area level (0.91 ± 16.59 cm^2^ vs. -7.1 ± 10.17 cm^2^, *P*=0.011), and subcutaneous fat area of abdomen level [0 (-18.75, 15.5) cm^2^ vs. -16.5 (-41.75, -2.25) cm^2^, *P*=0.014] were all greater in the Study group. (**Table 3**)

**Table 3:** Changes of blood sugar related indicators, physical measurements, and abdominal fat distribution.

Items	Study group (n=47)	Control group (n=38)	*P* value
Δ Fasting blood sugar (mmol/L)	-1.59 ± 3.45	-1.07 ± 2.11	0.432
Δ HbA1c (%)	-1.57 ± 2.00	-0.78 ± 1.03	0.025*
Δ Weight (kg)	-3.06 ± 4.15	-1.83 ± 3.83	0.166
Δ BMI (kg/m^2^)	-1.15 ± 1.51	-0.66 ± 1.41	0.136
Δ Waist circumference (cm)	-3.43 ± 7.07	-2.62 ± 5	0.553
Δ Hip circumference (cm)	-1.54 ± 6.91	-2.47 ± 23.35	0.800
Δ Waist to Hip ratio	-0.02 ± 0.06	-0.01 ± 0.07	0.680
Δ Fat mass (kg)	-1.82 ± 2.92	-1.74 ± 3.01	0.914
Δ Body fat ratio	-1.24 ± 1.65	-0.87 ± 3.12	0.516
Δ Visceral fat area (cm^2^)	-7.1 ± 10.17	0.91 ± 16.59	0.011*
Δ Abdominal cross-sectional area (cm^2^)	-17.98 ± 47.78	-24.08 ± 50.73	0.582
Δ Subcutaneous fat area of abdomen (cm^2^)	-16.5 (-41.75, -2.25)	0 (-18.75, 15.5)	0.014*
Δ Abdominal subcutaneous fat thickness (cm)	-0.1 ± 0.52	0.04 ± 0.54	0.240
Δ Back subcutaneous fat thickness (cm)	-0.04 ± 0.29	0.01 ± 0.48	0.630
Δ Subcutaneous fat thickness outside erector spinae (cm)	-0.16 ± 0.82	-0.01 ± 0.73	0.396
Δ Erector spinae intramuscular fat content (mg/cm^3^)	-0.70 (-42.98, 29.08)	13.56 (-40.59, 60.42)	0.422
Δ Liver fat content (%)	-2.11 (-4.65, 0.19)	-1.10 (-5.97, 1.95)	0.497

Data were expressed as mean± standard deviation and median (interquartile range).

HbA1c, hemoglobin A1c; BMI, Body Mass Index.

Δ = (endpoint level) - (baseline level).

*Compared with the Control group, P<0.05.

### Comparisons of the changes of surrogate biochemical markers of liver and kidney function between two groups

The changes in alanine aminotransferase (ALT), aspartate aminotransferase (AST), urea (BUN), serum creatinine (GRE), and urinary microalbumin clearance rate (UAER) between baseline and endpoint were compared. The differences were not statistically significant between the two groups (*P*>0.05). (**Table 4**).

**Table 4:** Changes in Liver and kidney function indicators.

Items	Study group (n=47)	Control group (n=38)	*P* value
Δ ALT (U/L)	-4 (-10, 1)	-8 (-20, 1.75)	0.725
Δ AST (U/L)	-1 (-10, 3)	-7.5 (-18.25, 1.25)	0.091
Δ BUN (mmol/L)	0.46 ± 1.46	0.15 ± 1.28	0.332
Δ CRE (µmol/L)	3.03 ± 13.38	-1.32 ± 7.18	0.203
Δ UAER(µg/min)	-4.83 ± 20.76	-3.19 ± 10.41	0.856

Data were expressed as mean± standard deviation and median (interquartile range).

UAER, urinary albumin excretion rates, CRE: creatinine; BUN, Blood urea nitrogen; AST, Aspartate aminotransferase; ALT, Alanine transaminase.

Δ = (endpoint level) - (baseline level).

### Adverse events in the two groups

In the Study group, there were 16 cases with nausea in the total of 47 cases, accounting for 34.04%; 3 cases with constipation, accounting for 6.38%; 3 cases with constipation, accounting for 6.38%, and no hypoglycemic events being reported. In the Control group, no hypoglycemic events were reported at the same time.

## Discussion

Obesity, especially abdominal obesity, is not only an important high-risk factor of T2DM but also closely related to cardiovascular complications, sleep apnea, and other combinations of T2DM (10). Excessive ectopic fat is more closely related to hyperinsulinemia, insulin resistance, dyslipidemia, and chronic inflammation, leading to a significant increase in the risk of cardiovascular events and sudden death (11). Therefore, weight management, especially to reduce excess abdominal fat load, is of great significance for patients with obesity and T2DM (12). Life interventions including diet control and exercise are important means of weight control, but it takes a long time to show the effect, and it is easy to rebound; meanwhile, studies have shown that exercise can increase muscle mass, but cannot reduce abdominal subcutaneous fat and visceral fat content (13). Metabolic surgery is a method for rapid weight loss and improvement of metabolism and visceral fat content; however, the surgery is an invasive treatment, and patients are concerned about the cost of surgery and the risks associated with surgery (14). Liraglutide is one of the GLP-1 analogs and is a new type of hypoglycemic drug that can bind to GLP-1 receptors to produce a variety of pharmacological effects, including promoting insulin secretion, reducing glucagon secretion, suppressing appetite, delaying gastrointestinal motility, and increasing the insulin sensitivity of muscle tissue (15). Studies have suggested that liraglutide intervention can reduce fat mass and preserve muscle tropism by improving blood lipids, glycemic control, and insulin sensitivity (16, 17). In this study, the GLP-1 receptor agonist liraglutide was applied to patients with high BMI, and compared with traditional lifestyle interventions, to study the effect of liraglutide on glucose metabolism and abdominal fat distribution in patients with obesity and T2DM. The results of this study showed that after using liraglutide, fasting blood glucose, HbA1c, body weight, BMI, waist circumference, fat mass, body fat ratio, visceral fat area, abdominal cavity area, abdominal subcutaneous fat area, and the liver fat content decreased significantly, and the statistically significant differences post-pre between the two groups were found just in HbA1c, Visceral fat, and Subcutaneous fat area of abdomen. Patients in the Control group who received lifestyle interventions had also experienced a decrease in fasting blood glucose, HbA1c, body weight, BMI, waist circumference, fat mass, and abdominal area, compared with the baseline level, and the difference within the group was statistically significant. But there were no significant differences in the abdominal subcutaneous fat area, liver fat content, and erector spine intramuscular fat content compared with the baseline level.

Several large-scale evidence-based studies such as the LEAD study, Lira–DPP-4i, and AMIGO have also confirmed that liraglutide can effectively improve blood sugar in patients with T2DM, whether alone or in combination with other hypoglycemic drugs (18). In the LEAD study, 1.8 mg/d liraglutide reduced glycosylated hemoglobin by 1.33%, similar to the results of this study, confirming that liraglutide can significantly improve blood glucose metabolism in patients with obesity and T2DM. Some scholars have initially explored the mechanism of liraglutide regulating fat metabolism and distribution *in vivo* and *in vitro*. Animal experiments found that after 12 weeks of liraglutide administration in male Wistar and Goto-Kakizaki rats, visceral fat decreased, subcutaneous fat increased, and the oxidative decomposition rate of fatty acids in skeletal muscle increased significantly (19). In addition, GLP-1 receptor agonists can regulate the differentiation of 3T3-L1 cells, a type of adipogenic precursor cells, through the serine-threonine kinase (AKT) signaling pathway, resulting in smaller adipocytes, which may contribute to insulin resistance and insulin resistance and have a positive effect in patients with obesity (20). In this study, it was found that the liver fat content in the Study group was significantly improved after the administration of liraglutide, which may be related to the following putative mechanisms: ① liraglutide may cause weight loss; ② liraglutide may regulate the M2 polarization of Kupffer cell; ③ liraglutide may decrease expression of NOD-like receptor thermal protein domain associated protein 3 (NLPR3) inflammasome; ④ liraglutide may improve the mitochondrial structure of hepatocytes and regulate autophagy; ⑤ liraglutide may regulate the local renin-angiotensin system (RAS) system in the liver (21–23). In this study, the reduction of liver fat content in the Study group was greater than that in the Control group, but there were no significant differences ​​between the two groups. Considering that it may be because of the same weight loss effect of the lifestyle intervention. In addition, some animal experiments have found that GLP-1 receptor agonists can reduce the fat content in the muscle of rats (24), but this study did not observe the reduction of the intramuscular fat content of the erector spine in the patients after the treatment of liraglutide, which may be related to the relatively small dose of liraglutide used in the study and the intervention period was short.

Weight, BMI, and waist circumference are diagnostic criteria for obesity and important indicators for judging the degree of obesity; ectopic fat refers to the fat contained in organs that are not physiological fat storage, such as the liver, skeletal muscle, pancreas, heart, etc. is an important cause of insulin resistance, chronic inflammatory state and is closely related with cardiovascular disease and metabolic disease (12). To evaluate the distribution of abdominal ectopic fat in patients with obesity and T2DM and study the body composition, DEXA and abdominal MRI methods were mostly used, but these methods cannot be widely used in clinical due to the high cost and long time required for examination (25). The energy spectrum CT quantification technology that appeared in recent years can perform density imaging of paired base substances through instantaneous high and low voltage switching, obtain the density ratio map of the paired base substances, use its attenuation change to analyze the content of base substances, and directly measure the content of abdominal visceral fat and intramuscular fat, and compared with MRI and DEXA, it has the advantages of shorter time consumption and lower radiation dose, which does not increase the X-ray radiation dose during the detection of fat content (5, 26). In this study, energy spectrum CT was innovatively used to evaluate the changes in abdominal fat distribution in patients with obesity and T2DM after liraglutide administration and lifestyle interventions. This study found that the visceral fat area and abdominal subcutaneous fat area of ​​the patients in the Study group were not only significantly lower than the baseline levels before medication but also decreased significantly more than that in the Control group, which was similar to with a retrospective study carried out by Satoshi Ishii et al. (27). In addition, this study also found that patients with obesity and T2DM in both the Study group and the Control group had significant improvements in body weight, BMI, and waist circumference compared with the baseline levels, and there were no significant differences in reductions in these indicators between the two groups. These findings suggested that lifestyle interventions were still effective in improving body mass and waist circumference in patients with obesity and T2DM. Therefore, this study confirmed that lifestyle interventions remain important means of managing patients with obesity and T2DM.

This study also has the following shortcomings: (1). The follow-up time was short, and the effect of liraglutide and its side effects might not be adequately studied; (2). There was a lack of quantitative assessment method to evaluate whether life interventions reached the set goal; (3). The relatively large number of people lost to follow-up in the Control group may cause selection bias.

## Conclusion

Both liraglutide and lifestyle intervention could reduce body weight, body fat content, and waist circumference in patients with obesity and T2DM; Compared with the lifestyle intervention, patients with the treatment of liraglutide may experience the more significant improvements in the changes of hemoglobin A1c, visceral fat area, and subcutaneous fat area of abdomen, and the incidence of adverse events of using the liraglutide was low. Liraglutide can be the drug choice for weight management and reduction of abdominal fat distribution in patients with obesity and T2DM.

## Data availability statement

The original contributions presented in the study are included in the article/**Supplementary Material**. Further inquiries can be directed to the corresponding author.

## Ethics statement

The studies involving human participants were reviewed and approved by Beijing Hospital, National Center of Gerontology. The patients/participants provided their written informed consent to participate in this study.

## Author contributions

DY and LG contributed to the conception and design of the study. MZ, QP, YZ, XW and YS performed the experiments, collected and analyzed data. DY, ML, XZ and LG wrote the manuscript. DY and LG revised the manuscript. All authors reviewed and approved the final version of the manuscript.

## Funding

This study was funded by National High Level Hospital Clinical Research Funding (No. BJ-2019-162).

## Conflict of interest

The authors declare that the research was conducted in the absence of any commercial or financial relationships that could be construed as a potential conflict of interest.

## Publisher’s note

All claims expressed in this article are solely those of the authors and do not necessarily represent those of their affiliated organizations, or those of the publisher, the editors and the reviewers. Any product that may be evaluated in this article, or claim that may be made by its manufacturer, is not guaranteed or endorsed by the publisher.
